# Emotion-gaze interaction affects time-to-collision estimates, but not preferred interpersonal distance towards looming faces

**DOI:** 10.3389/fpsyg.2024.1414702

**Published:** 2024-09-11

**Authors:** Daiki Yamasaki, Masayoshi Nagai

**Affiliations:** ^1^Research Organization of Open, Innovation and Collaboration, Ritsumeikan University, Osaka, Japan; ^2^Japan Society for the Promotion of Science, Tokyo, Japan; ^3^College of Comprehensive Psychology, Ritsumeikan University, Osaka, Japan

**Keywords:** time-to-collision, interpersonal distance, looming, emotion, facial expressions, gaze direction

## Abstract

Estimating the time until impending collision (time-to-collision, TTC) of approaching or looming individuals and maintaining a comfortable distance from others (interpersonal distance, IPD) are commonly required in daily life and contribute to survival and social goals. Despite accumulating evidence that facial expressions and gaze direction interactively influence face processing, it remains unclear how these facial features affect the spatiotemporal processing of looming faces. We examined whether facial expressions (fearful vs. neutral) and gaze direction (direct vs. averted) interact on the judgments of TTC and IPD for looming faces, based on the shared signal hypothesis that fear signals the existence of threats in the environment when coupled with averted gaze. Experiment 1 demonstrated that TTC estimates were reduced for fearful faces compared to neutral ones only when the concomitant gaze was averted. In Experiment 2, the emotion-gaze interaction was not observed in the IPD regulation, which is arguably sensitive to affective responses to faces. The results suggest that fearful-averted faces modulate the cognitive extrapolation process of looming motion by communicating environmental threats rather than by altering subjective fear or perceived emotional intensity of faces. The TTC-specific effect may reflect an enhanced defensive response to unseen threats implied by looming fearful-averted faces. Our findings provide insight into how the visual system processes facial features to ensure bodily safety and comfortable interpersonal communication in dynamic environments.

## 1 Introduction

Accurate perception of approaching or looming objects is crucial for organisms living in a dynamic three-dimensional environment. Looming stimuli increase in size on the retina over time, as a function of viewing distance, which induces a reliable perception of the approach. Previous studies have shown that the human visual system is highly sensitive to looming stimuli and prioritizes their processing (e.g., Cappe et al., 2012; Finlayson et al., 2012), yielding cognitive consequences such as attentional capture (Franconeri and Simons, 2003; Abrams and Christ, 2005) Looming, the specific visual pattern of expansion, results in increased neural activation in the visuomotor cortex (Peron and Gabbiani, 2009; Billington et al., 2011), and superior temporal sulcus (Tyll et al., 2013), seemingly leading to faster detection and motor responses to looming stimuli (Ball and Tronick, 1971; Yonas et al., 1979; Walker-Andrews and Lennon, 1985; Náñez and Yonas, 1994; Shirai and Yamaguchi, 2004; Lewkowicz, 2008). Avoiding collisions and maintaining an appropriate distance from others are commonly required in daily life and necessitates accurate spatiotemporal processing of looming individuals. Nevertheless, studies on looming perception in interpersonal situations are considerably scarce, and it has not been fully understood how the visual system monitors approaching others and what factors affect such processing.

In face-to-face interpersonal situations, the facial cues of an interaction partner are likely to influence the processing of looming others. Facial expressions that signal the expressor’s internal state are particularly important cues not only for social interactions with others but also for visual face processing. In particular, negative facial expressions are recognized more accurately (Macrae et al., 2002) and perceived as being closer compared to neutral expressions (Kim and Son, 2015), indicating their substantial impact of them on visual cognition. Furthermore, negative facial expressions serve as social threat cues that increase amygdala activity (Morris et al., 1996), which in turn triggers socioaffective responses such as avoidance behavior (Fox et al., 2007; Roelofs et al., 2010) and a greater interpersonal distance (Ruggiero et al., 2017, 2021; Silvestri et al., 2022). Thus, it is plausible to predict that negative facial expressions affect the spatiotemporal processing of looming faces.

Another factor that affects the processing of looming faces is gaze direction, which is an important cue for deducing the attentional focus of an interactive partner (for reviews, see de C Hamilton, 2016; Hadders-Algra, 2022) that also modulates and interpretation of emotional facial expressions (e.g., Sander et al., 2007). The effect of gaze direction on facial expression processing has also been found in face preference in newborns (Rigato et al., 2011), indicating an adaptive role of the emotion-gaze interaction in early age. The shared signal hypothesis posits that gaze direction differentially affects the perceived threats posed by negative expressions such as fear and anger, and that specific emotion-gaze pairs that convey a clear threat are particularly salient (Adams and Kleck, 2003, 2005). Specifically, fear indicates that the expresser perceives threats, which signals unseen threats in the environment when combined with an averted gaze. However, a direct gaze obscures the source of the threat and further communicates that the expressor is afraid of the observer. Anger, on the other hand, indicates aggression by the expressor, and when combined with direct gaze, suggests that the threat is directed at the observer. However, when combined with averted gaze, the target of threat becomes ambiguous. In line with this, it has been shown that the specific emotion-gaze pairs (i.e., fear-averted and anger-direct) elicit greater neuronal activity in the amygdala (Adams et al., 2003; Sato et al., 2004; N’Diaye et al., 2009). It has also been found that angry and happy faces with direct gaze and fearful faces with averted gaze are detected more accurately than the switched combinations, which helps observers orient their attention to environmental threats or rewards (Hietanen et al., 2008; Milders et al., 2011; de C Hamilton, 2016; McCrackin and Itier, 2019). The emotion-gaze pairs also affect emotional detection (Adams and Kleck, 2003, 2005), approach-avoidance tendencies (Roelofs et al., 2010), time perception (Doi and Shinohara, 2009; Kliegl et al., 2015), and gaze-cued attention (McCrackin and Itier, 2018a,b). Therefore, based on the shared signal hypothesis, we may predict that the emotion-gaze interaction affects the processing of looming faces.

When others are approaching, a crucial function of the visual system is to accurately estimate the time until impending collision, time-to-collision (TTC), based on visual inputs. Pioneering research theorized that TTC is estimated based on the visual variable *tau*, defined as the ratio of the retinal angle of a looming target to its instantaneous rate of optical expansion (tau theory, Lee, 1976; for a review, see Regan and Gray, 2000). However, subsequent studies have revealed that TTC estimates involve cognitive motion extrapolation (DeLucia and Liddell, 1998), decrease under a high cognitive load (Baurès et al., 2010; McGuire et al., 2021), and are affected by target size (DeLucia, 2004), initial distance (Yan et al., 2011), and velocity (Gray and Regan, 1998). Moreover, threatening targets (e.g., spiders, snakes, and frontal attacks) are estimated to arrive earlier than non-threatening targets (Brendel et al., 2012; Vagnoni et al., 2012), indicating an emotional impact. Furthermore, TTC estimates can decrease when participants’ movements are restricted, which constitutes a threatening situation given the difficulty of avoiding collisions (Neuhoff et al., 2012; Vagnoni et al., 2017). Despite accumulating findings suggesting that TTC estimation is not determined purely by physical optics, but also involves the processing of stimulus properties and threats in targets and the viewing environment, it remains unclear which factors affect the TTC estimation of looming people.

Aside from TTC estimation contributing to survival goals, maintaining an appropriate interpersonal distance (IPD) with approaching others is crucial for comfortable social interactions. In social psychology, IPD is considered to reflect personal space, in which individuals experience discomfort when others intrude (e.g., Hayduk, 1983), and plays an important role in social interactions. It has been suggested that individuals automatically adjust IPD based on the experienced discomfort and that IPD is regulated by an arousal-sensitive process involving the amygdala (e.g., Kennedy et al., 2009). Previous studies have shown that target individuals’ negative facial expressions and direct gaze of target individuals increase the preferred IPD (Bailenson et al., 2001; Asada et al., 2016; Ruggiero et al., 2017, 2021; Cartaud et al., 2018; Silvestri et al., 2022) by using a stop-distance task that measures the point at which observers experience discomfort with the approaching targets (e.g., Iachini et al., 2016; Silvestri et al., 2022). Although both negative facial expressions and direct gaze are known to increase the IPD, it is unclear whether the combination of these facial features interactively affects IPD regulation when being approached by others.

For our aim of examining TTC estimation and IPD regulation towards looming faces, it is meaningful to organize the concepts of the spatial regions associated with the judgments. Based on the finding that people with restricted body movements estimate shorter TTC of looming objects (Neuhoff et al., 2012; Vagnoni et al., 2017), reduced TTC estimates has been argued to reflect an enlargement of peripersonal space (PPS), the surrounding region serving as the spatial buffer for immediate actions (Rizzolatti et al., 1981, 1997; Di Pellegrino et al., 1997). On the other hand, preferred IPD is associated with a safety zone maintained for comfortable interpersonal interactions, which refers to a concept of social space that differs from the PPS as an action space (for a review: Coello and Cartaud, 2021). Recent studies have shown that both PPS and preferred IPD are altered by social relationships and emotions with interaction partners (Iachini et al., 2016; Patané et al., 2017), and that manipulations of reachability can alter the preferred IPD (Quesque et al., 2017; D'Angelo et al., 2019), albeit individual differences (Candini et al., 2019), suggesting that PPS and social IPD are functionally dissociable but share common spatial representations. Thus, note that TTC estimates and preferred IPD, which are similarly examined in spatiotemporal judgments towards approaching others, are considered to be related to different spatial concepts.

Previous studies have failed to demonstrate the effects of angry facial expressions on TTC estimates (Brendel et al., 2012; DeLucia et al., 2014). Note that Brendel et al. (2012) only found a reliable increase in TTC estimates for friendly faces relative to baseline empty faces, and observed an effect of threatening stimuli on TTC estimates when data from face and non-face images were pooled. Based on their results, it has been suggested that the TTC modulation requires unequivocal threats (i.e., predators) that elicit high arousal rather than implicit threats conveyed by facial expressions, which elicit less arousal (Wangelin et al., 2012). However, direct gaze by itself can affect visual cognition because it accompanies aggressive displays (Hinde and Rowell, 1962; Ioannou et al., 2014) and increases arousal, thus leading to cognitive consequences (Nichols and Champness, 1971; Mason et al., 2004; Senju and Hasegawa, 2005; Hietanen et al., 2008; Conty et al., 2010; Akechi et al., 2013; for a review, Emery, 2000). The lack of effect for angry facial expressions in previous studies may be attributable to the confounding effect of direct gaze paired with angry and neutral faces. Thus, for angry faces, which should convey threat along with direct gaze, the effects of facial expressions and direct gaze are inseparable, and the possible emotion-gaze interaction would be elusive. Therefore, we aimed to examine the emotion-gaze interaction effect using faces expressing fear as negative stimuli.

Therefore, the current study aimed to investigate whether fearful facial expressions and gaze direction have an interaction effect on the TTC estimation of and the preferred IPD from looming faces. Experiment 1 involved the TTC estimation task, which required participants to estimate the collision time after the looming faces disappeared, thus encompassing the spatiotemporal extrapolation of looming motion. Experiment 2 involved an IPD task that required participants to continuously observe looming faces and indicate when they felt discomfort. Given previous knowledge, it is hypothesized that fearful facial expressions reduce TTC estimates and increase preferred IPD for looming faces. The shared signal hypothesis further predicts that averted gaze enhances the influence of fearful facial expressions on the spatiotemporal processing of looming faces. On the other hand, the effect of direct gaze by itself, if any, was expected to appear only in the context of neutral faces, because the threat of fearful faces would be decreased by direct gaze.

## 2 Experiment 1

### 2.1 Method

#### 2.1.1 Participants

Sample size was determined using G*Power software (version 3.1.9.6; Faul et al., 2009) assuming an effect size (*η*_p_^2^ = 0.4) reported in a previous study that explored the effect of facial expressions on TTC estimates (Brendel et al., 2012). Based on the power analysis, a sample size of 15 participants were needed an interaction effect (α = 0.05, power = 0.8, ANOVA: fixed effects, special, main effects, and interactions). Therefore, in Experiment 1, we collected data from 15 students (10 females, *M* = 20.6 years, SD = 1.8). All participants reported normal or corrected-to-normal visual acuity and were naïve to the purpose of the study. In accordance with the Declaration of Helsinki, the participants signed a written consent form approved by the Ethics Review Committee for Research Involving Human Subjects of Ritsumeikan University.

#### 2.1.2 Apparatus and stimuli

Stimuli were generated using PsychoPy3 software (version 2021.1.4; Peirce et al., 2019) and presented on a black background on a 26-inch LCD screen (60 Hz, 1920 × 1,080 pixels; XL2411P; BenQ Desktop, Taipei, Taiwan), which was set at a viewing distance of approximately 57 cm. With their self-reported dominant eye, participants monocularly viewed the monitor in a standing position, ensuring veridical perception. The height of the monitor was individually adjusted to each participant’s eye level at the beginning of the experiment. Participants were required to maintain a standing position and keep their heads still during the experiment to ensure constant viewing conditions.

**Figure 1:**
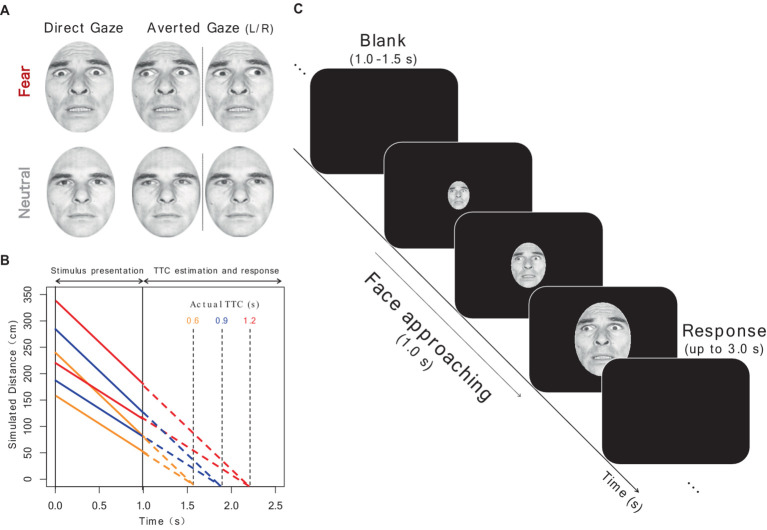
Schematic of experimental procedure. **(A)** Examples of fearful and neutral face stimuli with direct and averted gaze. Copyright is available in Ekman and Friesen (1976). Consulting Psychologists Press, Palo Alto, CA **(B)** Timeline of the approach of face stimuli. The simulated distance to the face (y-axis) is shown as a function of the time after stimulus onset (x-axis). Time during stimulus presentation and time after its disappearance are depicted by solid and dotted lines, respectively. Each actual TTC condition is indicated in different colors. **(C)** Timeline of a trial. After a blank, a face presented at the center of the screen expanded gradually over 1 s and then disappeared. Participants were required to extrapolate the motion and to press a key at the moment they thought the face would have collided with them. Source: “Pictures of Facial Affect (POFA)” © Paul Ekman 1993, https://www.paulekman.com/. Used with permission.

Figure 1 shows the face stimuli and procedures used in Experiment 1. The stimuli consisted of seven adult faces (four females and three males) selected from the Pictures of facial affect (Ekman and Friesen, 1976), each of which presented both neutral and fearful facial expressions.^1^ The gaze direction of each face was manipulated (direct gaze: 0°; averted gaze: leftward or rightward by 30°) using the Adobe Photoshop neural filter (version 22.5.1; Adobe, Mountain View, CA, United States). Each image was then superimposed by an oval mask (260 × 347 pixels), with the size and luminance held constant. In total, there were 28 face stimuli (7 faces×2 facial expressions×2 gaze directions).

We adopted three TTC conditions (actual TTC was simulated to be 0.6, 0.9, and 1.2 s), based on a previous study that investigated the effect of facial expressions on TTC (Brendel et al., 2012). To simulate a looming motion, the visual angle of the face stimulus was increased over 1 s (60 frames) and then it disappeared. The visual angle of the stimulus in each frame was simulated based on one of the three TTC conditions. If a face with a vertical length of *S* moves from a distance *D* at a constant velocity *v*, the subtended visual angle *θ* should follow the formula at time point *t*:


$$\theta_{t}=\frac{360}{\pi }\;\arctan\left(\frac{S}{2\left(D-V.t\right)}\right)$$

In the experiment, the visual angle was simulated by assuming a vertical face height of the face of 21 cm. The velocity of the stimulus was randomly varied (100 or 150 cm/s) to prevent participants from using a simple heuristic (Schiff et al., 1992), resulting in different initial and final distances.

#### 2.1.3 Procedure

Participants completed a TTC estimation task and following two subjective rating tasks. In the TTC estimation task, a face stimulus was presented at the center of the monitor and increased in size over 1 s, consistent with one of the three TTC conditions. Participants were instructed to mentally extrapolate the approaching face at a constant rate after its disappearance and to respond with a keypress at the moment when they judged that the face would have collided with them (prediction-motion task; DeLucia et al., 2014). A response was allowed for up to 3 s after the stimulus disappeared. The keypress latency in each trial was recorded as the estimated TTC. The next trial started automatically, followed by a random intertrial interval of 1–1.5 s. The TTC task consisted of 14 repetitions, in which each of the seven faces was presented twice, of 12 experimental conditions (2 emotions×2 gaze directions×3 TTC conditions), for a total of 168 trials. The experiment was divided into four blocks, each including two catch trials with TTCs of 0.5 and 1.5 s. Trial order was randomized across participants and blocks.

After the TTC estimation task, participants completed two subjective rating tasks using the same face stimuli presented in the TTC task. Participants observed a face stimulus and then indicated their degree of subjective fear (“How much did you fear the face?”), and the perceived intensity of the fear emotion expressed by the stimulus (“How strongly did the face express fear?”), on a 7-point Likert scale (1, not at all; 7, very much). The rating tasks of subjective fear and emotional intensity were conducted in separate blocks in a fixed order. Given the nature of looming motion as a signal of potential threat, we examined the possible influence of dynamic presentation on the ratings of face stimuli. Thus, in the rating tasks, 28 face stimuli (7 faces×2 facial expressions×2 gaze directions) were presented in looming and static forms, resulting in 56 trials. The stimuli in the looming condition were identical to those with a simulated TTC of 0.6 s and a velocity of 150 cm/s in the TTC task, consequently, they expanded from 5.01° to 13.31° over 1 s. In the static condition, the stimuli were presented for 1 s with a visual angle of 9.16°, corresponding to the average size in the looming condition. The stimuli were presented in a randomized order. To prevent participants from guessing the purpose of the TTC estimation task, the rating tasks were completed after the TTC task.

#### 2.1.4 Data analysis

Individual data of subjective ratings and TTC estimates were analyzed with linear mixed models using the *lme4* and *lmerTest* packages in R software (version 3.6.2; R Development Core Team, Vienna, Austria). The initial model included all possible fixed effects and by-participant random effects. Then, backward elimination was performed using the *step* function of the *lmerTest* package to identify the model that best explained the data. Backward elimination was conducted only for random effects, because a full-factorial analysis of variance provides test statistics for all fixed effects in the case of the balanced factorial designs (Barr et al., 2013; Matuschek et al., 2017). Based on the selected model, an *F*-test of the fixed effects was conducted using the *Anova* function of the *car* package. Since our primary aim was to investigate the interaction effect of facial expressions and gaze direction, we planned to conduct multiple comparisons across the four emotion-gaze conditions using the *difflsmeans* function of the *lmerTest* package, and *p* values were adjusted using the Holm method. The effect sizes were obtained using the *F_to_eta2* and the *t_to_d* functions in the *effectsize* package.

### 2.2 Results

#### 2.2.1 Effects of emotion and gaze direction on TTC estimation

**Figure 2:**
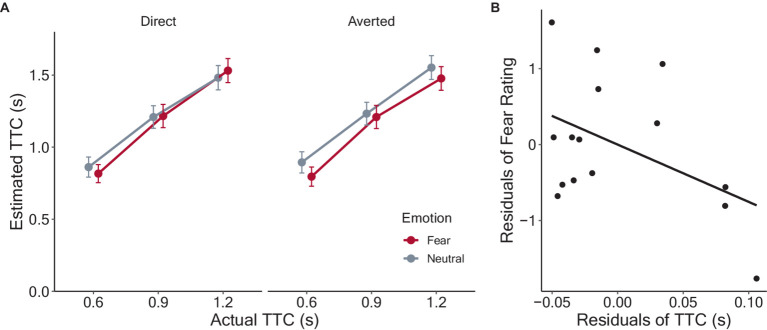
Results of the TTC estimation task. **(A)** Estimated TTC as a function of actual TTC in each condition. Each data point refers to the between-participant means with error bars showing 95% confidence intervals. Fear and neutral conditions are depicted in different colors. The dotted lines indicate veridical judgments of 0.6, 0.9, and 1.2 s. **(B)** Scatter plot showing relation of the residuals of TTC estimates and fear ratings.

Figure 2A shows the estimated TTC, which increased monotonically as a function of actual TTC. Trials in which no response was made within the time limit (2.3% of all trials) were excluded from the analyses. After applying backward elimination, the model included the main effects of emotion, gaze direction, and actual TTC and their interactions as fixed effects, in addition to random by-participant intercepts and slopes of the approaching velocity as random effects. The least-squares means of the TTC estimates for each condition were calculated, and the results are shown in Figure 2. The analysis revealed significant main effects of emotion (*F*(1, 2,378) = 3.91, *p* = 0.049, *η_p_^2^* = 0.002), due to smaller TTC estimates for fearful (M = 1.17 ± SD = 0.62) than neutral (1.20 ± 0.62). The main effect of actual TTC was also significant (*F*(1, 2,377) = 808.78, *p* < 0.001, *η_p_^2^* = 0.25), with smaller TTC estimates in the order of TTC_0.6 s_ (0.84 ± 0.50), TTC_0.9 s_ (1.22 ± 0.57), and TTC_1.2 s_ conditions (1.51 ± 0.59). However, the main effect of gaze direction was insignificant (averted: 1.19 ± 0.62; direct: 1.18 ± 0.62, *F*(1, 2,377) = 2.04, *p* = 0.153, *η_p_^2^* < 0.001). The interaction between emotion and gaze direction was significant (*F*(1, 2,377) = 3.98, *p* = 0.046, *η_p_^2^* = 0.002). However, the other two-way interactions were not significant (emotion and TTC: *F*(1, 2,377) = 0.95, *p* = 0.386, *η_p_^2^* < 0.001; gaze direction and TTC: *F*(1, 2,377) = 0.65, *p* = 0.524, *η_p_^2^* < 0.001). The three-way interaction among emotion, gaze direction, and TTC was not significant (*F*(1, 2,377) = 0.54, *p* = 0.586, *η_p_^2^* < 0.001).

Given the significant interaction between emotion and gaze direction, we conducted multiple comparisons to examine in more detail how these factors affected the TTC estimates. The mean TTC estimates and standard deviation were 1.16 ± 0.615 for fearful-averted, 1.19 ± 0.621 for fearful-direct, 1.22 ± 0.623 for neutral-averted, and 1.18 ± 0.612 for neutral-direct condition. The estimated TTC was significantly shorter for fearful-averted than that for neutral-averted condition (*t*(2377) = 2.80, *p* = 0.031, *d* = 0.11). However, there was no significant difference across other conditions (Fearful-averted vs. Fearful-direct: *t*(2377) = 0.40, *p* = 0.690, *d* = 0.02; Fearful-averted vs. Neutral-direct: *t*(2377) = 0.39, *p* = 0.697, *d* = 0.02; Fearful-direct vs. Neutral-averted: *t*(2377) = 0.01, *p* = 0.993, *d* = 0.002; Fearful-direct vs. Neutral-direct: *t*(2377) = 0.01, *p* = 0.064, *d* < 0.001; Neutral-averted vs. Neutral-direct: *t*(2377) = 2.42, *p* = 0.078, *d* = 0.10). Together, the analyses showed that fearful facial expressions led to shorter TTC estimates for approaching faces compared to neutral ones, but the effect emerged only when eye gaze was averted from the observer.

As previous studies have reported that people who have a greater fear of targets show greater reductions in the TTC estimates (Vagnoni et al., 2012, 2015, 2017), we examined whether TTC estimates were associated with participants’ reported fear ratings of the face stimuli. Following previous studies, to distinguish the effect of facial expressions from the variance related to individual differences, the individual means for fearful faces were regressed on those for neutral faces for both fear ratings and TTC estimates, and the residuals were obtained. The relationships between the residuals of the TTC estimates and fear ratings are shown in Figure 2B. A negative correlation between the residuals was observed, but it was not significant (*r* = −0.45, *p* = 0.09).

#### 2.2.2 Rating tasks: subjective fear and emotional intensity of the face stimuli

**Figure 3:**
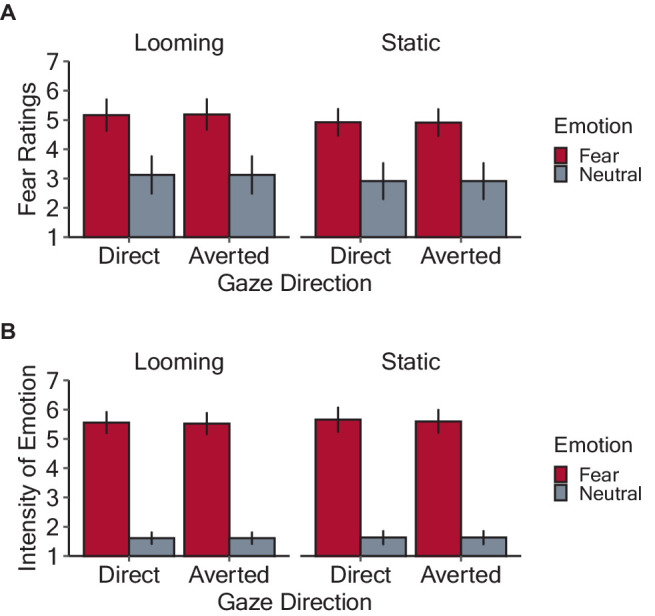
Results of the rating tasks in Experiment 1. **(A)** Mean fear rating of each experimental condition. **(B)** Mean rating for the perceived intensity of fear exhibited by the face stimuli. The bars denote the mean values with error bars showing 95% confidence intervals.

Figure 3A shows the mean ratings of the subjective fear induced by the stimuli. The model included the main effects of emotion, gaze direction, stimulus motion, and their interactions as fixed effects, as well as random by-participant intercepts. There were significant main effects of emotion (*F*(1, 802) = 371.46, *p* < 0.001, *η_p_^2^* = 0.32), due to higher scores for fearful (5.05 ± 1.34) than neutral (3.02 ± 1.63) condition, and of stimulus motion (*F*(1, 802) = 4.97, *p* = 0.026, *η_p_^2^* = 0.006), with higher scores for looming (4.11 ± 1.76) than static (3.88 ± 1.85) condition. However, the main effect of gaze direction was insignificant (averted: 3.04 ± 1.80; direct: 3.95 ± 1.81, *F*(1, 802) = 0.001, *p* = 0.97, *η_p_^2^* < 0.001). The interactions among these three factors were not significant (three-way interaction: *F*(1, 802) = 0.006, *p* = 0.94, *η_p_^2^* < 0.001; emotion and gaze direction: *F*(1, 802) = 0.001, *p* = 0.97, *η_p_^2^* < 0.001; emotion and stimulus motion: *F*(1, 802) = 0.06, *p* = 0.81, *η_p_^2^* < 0.001; gaze direction and stimulus motion: *F*(1, 802) = 0.005, *p* = 0.94, *η_p_^2^* < 0.001).

Figure 3B shows the mean ratings of the perceived intensity of fear emotion exhibited by the face stimuli in each condition. The selected model included the fixed effects of facial expression, gaze direction, stimulus motion, and all possible interactions, as well as the random by-participant intercepts. There was the main effect of emotion (*F*(1, 802) = 2442.26, *p* < 0.001, *η_p_^2^* = 0.75), due to higher scores for fearful (5.58 ± 1.27) than neutral (1.62 ± 0.995) condition. However, the main effects of gaze direction (averted: 3.59 ± 2.28; direct: d.46 ± 2.29, *F*(1, 802) = 0.07, *p* = 0.79, *η_p_^2^* < 0.001) and stimulus motion were not significant (looming: 3.50 ± 2.24; static: 3.55 ± 2.33, *F*(1, 802) = 0.42, *p* = 0.52, *η_p_^2^* < 0.001). There were no significant interactions (three-way interaction: *F*(1, 802) = 0.006, *p* = 0.94, *η_p_^2^* < 0.001; emotion and gaze direction: *F*(1, 802) = 0.07, *p* = 0.79, *η_p_^2^* < 0.001; emotion and stimulus motion: *F*(1, 802) = 0.18, *p* = 0.67, *η_p_^2^* < 0.001; gaze direction and stimulus motion: *F*(1, 802) = 0.005, *p* = 0.94, *η_p_^2^* < 0.001).

The results indicate that the emotional valence of the face stimuli was successfully manipulated. Looming faces were rated as more threatening than static faces, whereas the intensity of emotional facial expressions did not differ between looming and static faces. Given that looming motion serves as a warning signal to avoid a possible collision (e.g., Neuhoff, 2001; Bach et al., 2008; Cappe et al., 2012), it is intuitive that such a looming bias was observed for fear ratings but not for the perception of facial expression.

### 2.3 Discussion

Experiment 1 examined whether fearful facial expressions interacted with gaze direction in the TTC estimates of looming faces. We demonstrated that fearful faces were judged to arrive earlier than neutral faces only when the faces’ gaze was averted, indicating that fear reduced TTC estimates depending on the concomitant gaze. This result is consistent not only with previous findings that negative stimuli can reduce TTC estimates (e.g., Vagnoni et al., 2012), but also with the prediction based on the shared signal hypothesis, which assumes that fearful facial expressions should signal clear threats when coupled with averted gaze, providing new evidence of the susceptibility of TTC estimation to negative facial features.

Subjective ratings showed that the fearful faces were perceived to be expressing more intense fear and induced more fear than neutral faces, confirming that fear valence was successfully manipulated. However, the ratings varied based solely on facial expressions, regardless of gaze direction, unlike the TTC estimation. The gaze effect has been shown to occur only when the emotional expression is ambiguous for emotion intensity judgments (N’diaye et al., 2009) and for emotion discrimination (Graham and LaBar, 2007). Considering that our fearful faces clearly expressed fear, as indicated by the rating tasks, the insignificant gaze effect might be due to the manifest emotion in the stimuli, at least in consciously experienced emotions.

In addition, given the non-significant correlation between subjective fear and TTC reduction, it is unlikely that fear of the faces directly affected the TTC estimates. What factor could have reduced the TTC estimates for looming faces? According to the previous studies, fearful faces are not dangerous *per se* but imply the existence of an impending peril, which, coupled with averted gaze, induces environmental threats (e.g., Sander et al., 2007; Adams et al., 2012). Thus, one plausible explanation is that the TTC estimates were reduced owing to threats related to the viewing environment implied by the fearful-averted faces. Indeed, visual processing can change in threatening viewing contexts (Righart and de Gelder, 2008; Stefanucci et al., 2012; Wieser and Brosch, 2012; Tabor et al., 2015; Stolz et al., 2019). TTC estimates have also been shown to decrease in threatening situations (Neuhoff et al., 2012; Vagnoni et al., 2017). Against this background, it is feasible that environmental threats signaled by fearful-averted faces, rather than the specific fear of looming stimuli (Brendel et al., 2012; Vagnoni et al., 2012; DeLucia et al., 2014), reduced the TTC estimates in this experiment.

However, the reduced TTC estimates might reflect affective responses to the faces rather than visual processing of the looming motion. A potentially confounding factor would be discomfort with looming faces because negative facial expressions and direct gaze have been shown to increase preferred IPD from others (Bailenson et al., 2001; Asada et al., 2016; Ruggiero et al., 2017; Cartaud et al., 2018; Ruggiero et al., 2021; Silvestri et al., 2022), which seems to be adjusted according to the degree of discomfort experienced (e.g., Kennedy et al., 2009). Therefore, using the same face stimuli, Experiment 2 examined the emotion-gaze interaction in the preferred IPD task, where participants were asked to respond when they felt discomfort when viewing looming faces (e.g., Iachini et al., 2016; Silvestri et al., 2022). If the result of the TTC estimation task was due to increased discomfort with the fearful-averted faces, a similar interaction should be observed in the affective judgments of IPD from looming faces.

## 3 Experiment 2

### 3.1 Method

#### 3.1.1 Participants

A different group of 15 students (six females, *M* = 21.9 years, *SD* = 2.43) with normal or corrected-to-normal vision participated in the experiment. Since there was no prior assumption on the effect size of the emotion-gaze interaction on preferred IPD judgments, we determined to collect the same sample size as in Experiment 1. All participants were naïve to the purpose of the experiment. All participants signed a written consent form in accordance with the Declaration of Helsinki, and the study was approved by the Ethics Review Committee for Research Involving Human Subjects of Ritsumeikan University.

#### 3.1.2 Apparatus, stimuli, and procedure

The same apparatus and stimuli used in Experiment 1 were employed in Experiment 2, except that the visual angle was increased from 3° to 36° over 3 s, thereby simulating a face approaching the observer from a distance of 390 cm to 30 cm at a constant speed. Participants performed the preferred IPD task (e.g., Silvestri et al., 2022), where they were asked to press a key when they felt that the faces violated their personal space. They were instructed to respond as soon as they felt that the distance between them and the face stimuli made them uncomfortable. Unlike Experiment 1, where faces disappeared during the trial, in Experiment 2, the stimulus was presented until response and disappeared following the response or when no keypress was detected for 4 s after stimulus onset. A shorter response time indicated that the face caused discomfort at a greater distance from the observer. All four experimental conditions (2 emotions×2 gaze direction) were presented 21 times for a total of 84 trials (separated into two blocks). Trial order was randomized across participants and blocks.

Participants then completed two blocks of the subjective rating tasks, which were the same as those used in Experiment 1. Given that stimulus motion showed no interaction with emotion and gaze direction factors in Experiment 1, only the looming condition was examined herein. The rating tasks were again notified after the main task to prevent participants from ascertaining the purpose of the study.

### 3.2 Results

#### 3.2.1 Effects of emotion and gaze direction on the preferred IPD

**Figure 4:**
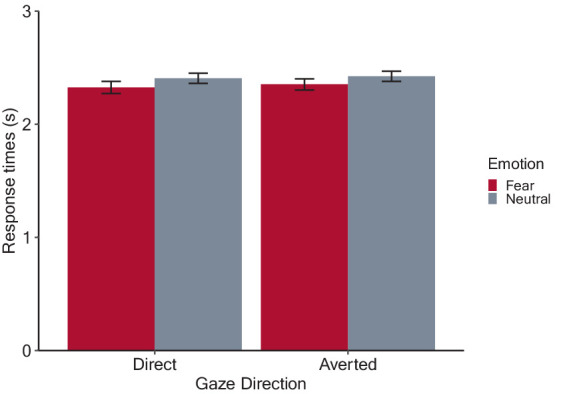
Results of the preferred interpersonal distance (IPD) judgment task. The bars denote mean response times with error bars showing 95% confidence intervals. Shorter response times indicate that participants experienced discomfort at a greater simulated IPD from looming faces.

Figure 4 shows the estimated mean response times from stimulus onset for each condition. After applying backward elimination, we obtained a linear mixed model including the main effects of emotion and gaze direction and their interaction as fixed effects, as well as the random by-participant intercepts. The analysis revealed a significant main effect of emotion (*F*(1, 1,276) = 24.88, *p* < 0.001, *η_p_^2^* = 0.02), due to shorter response times for fearful (2.34 ± 0.47) than neutral (2.42 ± 0.41) condition. However, the main effect of gaze direction (averted: 2.39 ± 0.44; direct: 2.37 ± 0.46, *F*(1, 1,276) = 1.19, *p* = 0.275, *η_p_^2^* < 0.001), and the interaction between emotion and gaze direction were not significant (*F*(1, 1,276) = 0.32, *p* = 0.570, *η_p_^2^* < 0.001). These results indicate that fearful facial expressions decreased response times from the onset of looming faces, which indicates that participants maintained a greater IPD from fearful compared to the neutral faces, but the emotional effect remained regardless of gaze direction.

#### 3.2.2 Rating tasks

**Figure 5:**
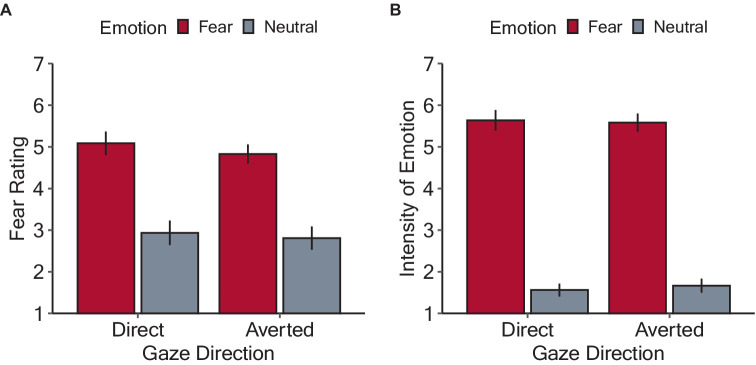
Results of the rating tasks in Experiment 2. **(A)** Mean fear rating for each experimental condition. **(B)** Mean perceived intensity of the fear expressed by the face stimuli. The bars denote mean values with error bars showing 95% confidence intervals.

Figure 5 shows the mean subjective fear ratings and perceived intensity of fear expressed by the face stimuli. Regarding the subjective fear ratings, a linear mixed model including the by-participant random intercepts revealed a significant main effect of emotion (*F*(1, 402) = 362.72, *p* < 0.001, *η_p_^2^* = 0.47), with higher scores for fearful (4.91 ± 1.04) than neutral (2.88 ± 0.99) condition. The main effect of gaze direction was not significant (averted: 3.80 ± 1.34; direct: 4.00 ± 1.54, *F*(1, 402) = 3.03, *p* = 0.083, *η_p_^2^* = 0.007). There was no significant interaction between emotion and gaze direction (*F*(1, 402) = 0.37, *p* = 0.543, *η_p_^2^* < 0.001). Regarding the perceived intensity of fear expressed by the face stimuli, a linear mixed model including the by-participant random intercepts revealed a significant main effect of emotion (*F*(1, 402) = 1955.74, *p* < 0.001, *η_p_^2^* = 0.83), with higher scores for fearful (5.61 ± 0.77) than neutral (1.61 ± 0.35) condition. However, the main effect of gaze direction was not significant (averted: 3.62 ± 2.97; direct: 3.60 ± 2.17, *F*(1, 402) = 0.07, *p* = 0.371, *η_p_^2^* < 0.001). There was no significant interaction between emotion and gaze direction (*F*(1, 402) = 0.80, *p* = 0.079, *η_p_^2^* = 0.002). These results indicate that the emotions expressed by the facial stimuli were satisfactorily manipulated and were robust against the effects of gaze direction.

### 3.3 Discussion

The results of the rating tasks were highly consistent with those in Experiment 1. Faces with fearful expressions were rated as expressing and inducing more fear than neutral ones, indicating that fear valence was successfully manipulated. Again, the ratings were not modulated by gaze direction, replicating the results of Experiment 1. In the IPD task, we found that participants stopped the faces earlier for fearful faces compared to neutral faces, which indicates that an increased preferred IPD from fearful faces, consistent with the previous knowledge of negative facial expression effects (Ruggiero et al., 2017, 2021; Cartaud et al., 2018; Silvestri et al., 2022). However, there was no gaze effect or the emotion-gaze interaction, which is inconsistent with that direct gaze can also increase preferred IPD (e.g., Bailenson et al., 2001). The consistent results for IPD, which is thought to be sensitive to affective responses to the target, and the rating tasks that assess conscious emotions toward the faces suggests that fearful facial expressions alter emotional responses to faces, resulting in the maintenance of a greater distance to looming faces expressing fear. On the other hand, given that no emotion-gaze interaction was found in either the IPD or the rating tasks, it is unlikely that gaze direction affected the emotion processing of the looming faces.

The lack of an effect of direct gaze on IPD could be at least partly due to the study methodology. While a direct gaze effect on IPD was evident in previous experiments involving real dyads or realistic virtual avatars, no such effect was found in experimental settings where printed pictures of faces were used (Sicorrelo et al., 2019). Less realistic faces with direct gaze have been shown to evoke weaker neural responses in the amygdala, while responses to facial expressions do not depend on facial realism (Pönkänen et al., 2011; Kätsyri et al., 2020). Moreover, direct gaze enhances the skin conductance response (SCR), a reliable measure of arousal (e.g., Nichols and Champness, 1971; Strom and Buck, 1979; Pönkänen and Hietanen, 2012), albeit only when engaging in a demanding task (Conty et al., 2010). Considering the nature of our task, which required participants to make a simple keypress when discomfort was felt in response to gray-scaled faces, direct gaze might have elicited insignificant increases in arousal, which is responsible for regulating preferred IPD (Kennedy et al., 2009). However, using the same task as ours, Silvestri et al. (2022) found that adult participants maintained a greater IPD from angry faces than happy and neutral faces, with no change in SCR by facial emotions. This suggests that preferred IPD may be susceptible to facial emotion, independent of physiological emotional responses.

Ambiguity in the interpretation of direct gaze might have attenuated the gaze effect, given that direct gaze may be associated with both hostility and intimacy (e.g., Argyle et al., 1986; Akechi et al., 2013; Hessels et al., 2017; Cui et al., 2019), which predict an increase and decrease in IPD, respectively. Additionally, the gaze effect on face processing depends inversely on the intensity of facial expressions (Graham and LaBar, 2007; N’diaye et al., 2009; Caruana et al., 2019), whereas the processing of emotions seems to be preconscious (Öhamn et al., 2001; Jiang et al., 2009; Stins et al., 2011; for a review, Frischen et al., 2008). Given the results of the rating tasks, which repeatedly indicated that our stimuli expressed manifest fear, it is possible that gaze direction had little effect on the affective processing of the fearful faces in this study. Together, Experiment 2 showed that the preferred IPD to looming faces was increased by clearly expressed fearful expressions but not by gaze direction.

This study was limited to examining a situation where participants passively observe looming faces, leaving the emotion-gaze interaction in IPD regulation during active approach unclear. Preferred IPD has been known to be greater when people are approached passively by others than when they approach others actively (Iachini et al., 2016). It is possible that people prefer to maintain a smaller IPD to others in situations where the existence of threats is implied, which is an intriguing hypothesis for future research. Additionally, since the sample size was calculated based on the effect size of facial expressions on TTC estimates in a previous report (Brendel et al., 2012), the result of null interaction effect in preferred IPD may have been underpowered. Although we analyzed the data designed for an ANOVA using mixed models, it has been suggested that a Monte Carlo simulation is effective for the sample size calculation for mixed models (Green and Mac Leod, 2016), which is another limitation of this research and should be considered in future research.

## 4 General discussion

We investigated the interaction effect of fearful facial expressions and gaze direction on TTC estimation and preferred IPD judgments toward looming faces, according to the shared signal hypothesis that fearful facial expressions with averted gaze strongly affect TTC estimates of looming faces (Adams and Kleck, 2003, 2005). Experiment 1 demonstrated the hypothesized emotion-gaze interaction on TTC estimates. Specifically, fearful faces led to shorter TTC estimates than neutral faces only when the gaze was averted from participants and disappeared when the gaze was directed toward them. In Experiment 2, participants displayed a greater preferred IPD for fearful faces than for neutral faces, regardless of gaze direction. Considering that the IPD is adjusted according to degree of discomfort and is regulated by an automatic arousal-sensitive process (e.g., Kennedy et al., 2009), the effect of emotion-gaze interaction on the TTC estimates observed herein is unlikely to be due to affective processes such as increased arousal, discomfort, or avoidant responses to the looming faces themselves. Additionally, an interaction between fearful facial expressions and gaze direction was observed in the TTC task, in which the looming stimuli disappeared during the trial, but not in the IPD task, in which the stimuli were presented until response. Given the different stimuli presentation procedures between the two tasks, our results suggest that integrated facial cues affect the cognitive extrapolation processing of unseen looming motion, which is only required in the TTC estimation task (DeLucia and Liddell, 1998; DeLucia, 2004). Taken together, these results indicate that fearful facial expressions interact with gaze direction in the spatiotemporal judgment of TTC, but not in the socioaffective judgment of preferred IPD from looming faces.

To the best of our knowledge, this is the first observation of an underestimation effect on TTC estimates by fearful facial expressions, although it has been reported that friendly faces increased TTC estimates compared to baseline empty faces (Brendel et al., 2012). Previous TTC studies that did not detect such an effect used only direct gaze stimuli (Brendel et al., 2012; DeLucia et al., 2014), which may have confounded the effects of facial expressions and direct gaze. Thus, the effects of facial expressions and the emotion-gaze interaction were uncertain. In contrast, the current study isolated the effects of negative facial expressions and direct gaze by examining fearful facial expressions, which should have a stronger influence with averted gaze. Given that fearful faces, unlike dangerous animals and angry confederates used in previous studies, are not themselves threatening targets, the observed reduction in TTC estimates by fearful-averted faces is unlikely to be due to increased fear of the stimuli, as suggested by the rating tasks. Based on previous studies showing that fearful faces coupled with averted gaze signal the existence of threats in the surrounding environment (Adams and Kleck, 2003, 2005; Adams et al., 2012), our results may be explained in terms of the environmental threats signaled by the faces, in line with the fact that TTC estimates decrease in situations where defensive actions are restricted (Neuhoff et al., 2012; Vagnoni et al., 2017). Thus, our findings suggest the susceptibility of TTC estimation to the environmental threats implied by the combination of facial features, in addition to the fear of the targets themselves (Brendel et al., 2012; Vagnoni et al., 2012; DeLucia et al., 2014). Note that the reduced TTC estimates in the averted gaze condition can hardly be explained by changes in arousal or face processing due to direct gaze (e.g., Nichols and Champness, 1971; Strom and Buck, 1979; Pönkänen and Hietanen, 2012). Given the previous knowledge of the beneficial role of mutual gaze in collision avoidance behavior (Nummenmaa et al., 2009; Narang et al., 2016), the prediction of collisions with looming faces might have been increased by averted gaze, which indicates that the expressor is unaware of the observer, thus affecting TTC estimates. However, if the attentional focus of faces inferred from gaze direction affects TTC estimation, a similar effect should also have appeared in the neutral expression condition.

The TTC task in Experiment 1 may have involved representations of faces in considerable proximity to observers or even collisions with faces, whereas the stimuli were stopped before intruding into participants’ personal space in Experiment 2. The TTC-specific modulation may be related to the intrusion of faces into the peripersponal space, or PPS (Rizzolatti et al., 1981; Di Pellegrino et al., 1997; Rizzolatti et al., 1997), and to an enlargement of PPS to prepare defensive responses to looming faces posing a threatening context (Neuhoff et al., 2012; Vagnoni et al., 2017). It has been known that the boundary of PPS is modulated by threats related to viewing situations (Lourenco et al., 2011; Sambo and Iannetti, 2013; Bufacchi and Iannetti, 2018; Serino, 2019), and that the defensive function of PPS is enhanced by fearful faces presented nearby (Ellena et al., 2020, 2021). Given these findings, a possible speculation based on our results is that in the TTC estimation task, the representational intrusion of faces into participants’ PPS may have enhanced defensive responses to environmental threats implied by fearful-averted faces.

Given the similarities between PPS and IPD (Iachini et al., 2016; Ruggiero et al., 2017; Patané et al., 2017; Quesque et al., 2017; D'Angelo et al., 2019), one might expect the effects observed in TTC estimation to also appear in IPD regulation. Although we demonstrated that the emotion-gaze interaction affects TTC estimates but not preferred IPD, we did not examine whether these two spatiotemporal judgments vary in tandem within individuals. It is intriguing to address the possibility that responses of PPS and IPD to face-induced threats may be dissociated in future studies. Another limitation of this study is that a possible influence of gender was not examined. Emotional facial expressions have been found to shrink preferred IPD of male more than female participants (Silvestri et al., 2022), raising a question whether the emotion-gaze interaction in the processing of looming faces show gender differences. Our post-hoc analysis of the effect of participant gender on preferred IPD preliminarily found that females (2.09 ± 0.34) showed faster response times than males (2.56 ± 0.40), without any interactions with emotions and gaze direction (data not shown). This is consistent with the knowledge that females perceive approaching stimuli as more intruding than males (Wabnegger et al., 2016), although inconsistent with Silvestri et al. (2022) showing faster response times for males than females. Moreover, it has been known that when participants passively observe approaching others, judgments of reachable and comfortable distances are both larger for male than female confederates (Iachini et al., 2016). Given the effect of stimulus gender in the judgments related to PPS and IPD, it is possible that stimulus gender affected TTC estimates and preferred IPD, but our post-hoc analyses found no effect of stimulus gender in both tasks (data not shown), possibly due to the gray-colored and cropped face stimuli and the modest sample size. Therefore, the influences of the gender of participants and stimulus, if any, were not crucial for interpreting our results in this study. However, future studies could include these as possible factors that may influence the emotion-gaze interaction in the processing of looming faces.

There were some potential confounders in this study, none of which undermined the results. First, since tau theory originally postulates that TTC estimates depend on the rate of optical expansion, the decrease in TTC estimates could have been related to perceptual modulations. Because threatening targets and viewing situations can cause observers to overestimate size and underestimate distance (e.g., van Ulzen et al., 2008; Stefanucci et al., 2012; Vasey et al., 2012; Leibovich et al., 2016), it is possible that the fearful-averted faces were perceived as larger or closer than actual, both of which would predict decreased TTC estimates. Such perceptual modulations could have appeared in the IPD task, where visual inputs provided until response would have yielded a continuous perception of looming faces, but this was not the case. Thus, the effect of emotion-gaze interaction on TTC estimates is unlikely to be due to ongoing changes in visual perception. Second, the stimulus duration differed between tasks. While the stimuli were presented for 1 s and then disappeared in Experiment 1, in Experiment 2, they were displayed for longer periods (average, 2.4 s) until a response. The shorter duration of stimuli presentation and smaller optical size in the TTC task may have reduced emotional discriminability, which underlies the gaze effect in emotion judgment tasks (Graham and LaBar, 2007; N’diaye et al., 2009). However, given the rapid processing of facial expressions observed in electroencephalography studies (Eimer and Holmes, 2002; Schyns et al., 2007; Poncet et al., 2019), a duration of 1 s seems sufficient for recognizing facial expressions, as indicated by our rating tasks. Additionally, the simulated velocities of the looming stimuli were similar in the two experiments, precluding any influence of motion velocity. Third, it is worth mentioning that the perceived duration of angry faces has been known to be lengthened by increased arousal (e.g., Doi and Shinohara, 2009; Gil and Droit-Volet, 2012; Kliegl et al., 2015). If the perceived duration of the fearful-averted faces had been lengthened, such an effect might also have been observed in preferred IPD judgments, which are sensitive to arousal. In addition, a longer stimulus duration should result in a lower rate of optical expansion within a unit of time, which would predict increased TTC estimates. Finally, the stimuli consisted of faces of other races from that of our Japanese participants. Although it is known that facial ethnicity can influence the emotional processing of facial expressions (for a review, Elfenbein and Ambady, 2002), the rating tasks confirmed accurate emotion perception in both experiments. Therefore, the other-race effect is unlikely to be crucial to our finding of emotion-gaze interaction on TTC estimates. Other-race faces with direct gaze can increase amygdala responses more than own-race faces (Richeson et al., 2008). Moreover, amygdala responses to fearful expressions were shown to be larger for averted gaze in own-race faces, but for direct gaze in other-race faces (Hadjikhani et al., 2008; Adams et al., 2010). Also, the fearful-averted bias for own-race faces can disappear in Japanese participants (Adams et al., 2010), likely due to the perception of direct gaze as intrusive in Asian cultures (Graham and LaBar, 2012; Akechi et al., 2013). Since all participants in this study were Japanese, the fearful-direct gaze in the other-race face stimuli might have increased amygdala activity. However, our results did not show a specific effect of fearful-direct faces, even in preferred IPD involving amygdala activity (Kennedy et al., 2009). Future studies could examine the mechanism of the observed emotion-gaze interaction in more detail through inter-racial experiments using brain imaging techniques and physiological measures.

The current study demonstrated that fearful facial expressions and gaze direction interact on TTC estimation, but not on preferred IPD from looming faces, providing important insights into how the visual system processes and monitors looming motion to ensure bodily safety and comfortable interpersonal communication. Our findings show that TTC estimation is susceptible to a specific combination of facial features. TTC-specific modulation by fearful-averted faces can be interpreted in terms of environment-related threats rather than affective responses to the faces themselves. Reduced TTC estimates in situations in which a threat is implied could reflect an adaptive bias of the visual system to prepare a safety margin for avoiding collisions. On the other hand, the finding that direct gaze regulates the effect of fearful facial expressions suggests that mutual gaze may preclude the emotional impact on the veridical perception of looming motion. This is consistent with the previously known benefit of mutual gaze in collision avoidance (Nummenmaa et al., 2009; Narang et al., 2016), reconciling adaptive bias with the beneficial role of mutual gaze in interpersonal communication. The limitations of this study are that we only investigated fearful facial expressions and did not measure face-induced environmental threats. Future studies need to examine other facial expressions and use physiological measures and behavioral tasks related to defensive responses to invisible threats to better understand the detailed characteristics and mechanisms of the emotion-gaze interactions in the perceptual, cognitive, and emotional processing of looming individuals.

## Data availability statement

The raw data supporting the conclusions of this article will be made available by the authors, without undue reservation.

## Ethics statement

The studies involving humans were approved by Ethics Review Committee for Research Involving Human Subjects, Ritsumeikan University. The studies were conducted in accordance with the local legislation and institutional requirements. The participants provided their written informed consent to participate in this study.

## Author contributions

DY: Conceptualization, Data curation, Formal analysis, Funding acquisition, Investigation, Methodology, Project administration, Resources, Writing – original draft, Writing – review & editing. MN: Conceptualization, Supervision, Writing – review & editing.

## Footnotes

^1^Note that all participants were Japanese, and their ethnicity differed from that of Caucasian face stimuli. While an own-race advantage has been shown in face perception (e.g., Elfenbein and Ambady, 2002), it has also been reported that Asian participants exhibit a low accuracy when categorizing fearful expressions and tend to misclassify Asians’ facial expressions as sad (Jack et al., 2009; Ma et al., 2022). Given our primary aim of examining the combination of fearful expressions and averted gaze, a well-validated dataset was used to ensure veridical perception of fear.
